# (2*E*,6*E*)-2,6-Bis(ferrocenyl­methyl­idene)cyclo­hexa­none dichloro­methane monosolvate

**DOI:** 10.1107/S1600536812030899

**Published:** 2012-07-14

**Authors:** Shi-Jia Long, Wu Yang, Yong-Hong Liu

**Affiliations:** a College of Life Science and Chemistry, Tianshui Normal University, Tianshui 741001, People’s Republic of China; b College of Chemistry and Chemical Engineering, Northwest Normal University, Lanzhou 730070, People’s Republic of China; c College of Chemistry and Chemical Engineering, Yangzhou University, Yangzhou 225002, People’s Republic of China

## Abstract

In the title compound, [Fe_2_(C_5_H_5_)_2_(C_18_H_16_O)]·CH_2_Cl_2_, the C=C bonds both adopt *E* conformations. In one ferrocenyl group, the five-membered rings are in a near-eclipsed conformation, whereas in the other they are mutually rotated by *ca* 21.5°. The central cyclo­hexa­none ring adopts a sofa conformation. In the crystal, the dichloro­methane solvent moleucle forms C—H⋯O hydrogen bonds to the organometallic mol­ecules to generate [010] chains of alternating solvent and organometallic species.

## Related literature

For our ongoing research in this area, see: Long *et al.* (2008 ▶); Liu & Guo (2010 ▶); Liu *et al.* (2008 ▶). For synthesis, see: Bai *et al.* (2004 ▶).
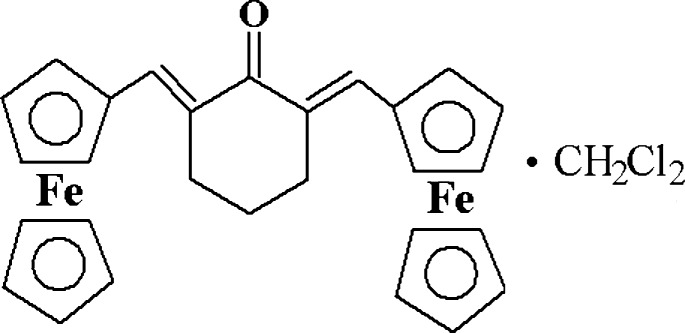


## Experimental

### 

#### Crystal data

[Fe_2_(C_5_H_5_)_2_(C_18_H_16_O)]·CH_2_Cl_2_
*M* *_r_* = 575.11
Monoclinic, 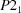

*a* = 9.417 (4) Å
*b* = 9.330 (4) Å
*c* = 14.449 (6) Å
β = 100.127 (5)°
*V* = 1249.7 (8) Å^3^
*Z* = 2
Mo *K*α radiation
μ = 1.40 mm^−1^
*T* = 296 K
0.42 × 0.16 × 0.14 mm

#### Data collection

Bruker SMART CCD diffractometer
Absorption correction: multi-scan (*SADABS*; Sheldrick, 2008*a* ▶) *T*_min_ = 0.592, *T*_max_ = 0.829
6984 measured reflections
4224 independent reflections
2923 reflections with *I* > 2σ(*I*)
*R*_int_ = 0.036

#### Refinement

*R*[*F* ^2^ > 2σ(*F* ^2^)] = 0.052
*wR*(*F* ^2^) = 0.127
*S* = 1.00
4224 reflections
307 parameters
1 restraint
H-atom parameters constrained
Δρ_max_ = 0.40 e Å^−3^
Δρ_min_ = −0.49 e Å^−3^
Absolute structure: Flack (1983 ▶), with 1883 Friedel pairs
Flack parameter: 0.60 (4)

### 

Data collection: *SMART* (Bruker, 2007 ▶); cell refinement: *SAINT* (Bruker, 2007 ▶); data reduction: *SAINT*; program(s) used to solve structure: *SHELXS97* (Sheldrick, 2008*b* ▶); program(s) used to refine structure: *SHELXL97* (Sheldrick, 2008*b* ▶); molecular graphics: *PLATON* (Spek, 2009 ▶); software used to prepare material for publication: *SHELXTL* (Sheldrick, 2008*b* ▶).

## Supplementary Material

Crystal structure: contains datablock(s) I, global. DOI: 10.1107/S1600536812030899/hb6805sup1.cif

Structure factors: contains datablock(s) I. DOI: 10.1107/S1600536812030899/hb6805Isup2.hkl

Additional supplementary materials:  crystallographic information; 3D view; checkCIF report

## Figures and Tables

**Table 1:** Hydrogen-bond geometry (Å, °)

*D*—H⋯*A*	*D*—H	H⋯*A*	*D*⋯*A*	*D*—H⋯*A*
C1*S*—H1*S*1⋯O1	0.97	2.46	3.210 (9)	133
C1*S*—H1*S*2⋯O1^i^	0.97	2.25	3.098 (9)	145

Symmetry code: (i) 
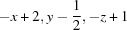
.

**Table 2:** Dihedral angles (°) for selected planes

	Atoms defining plane	1-Plane	Cps1-Plane	Cp1-Plane	Cp2-Plane
1-Plane	O1/C12/C16/C17				
Cps1-Plane	C19–C23	11.2 (4)			
Cp1-Plane	C24–C28	10.5 (6)	1.2 (6)		
Cp2-Plane	C1–C5	19.4 (5)	9.3 (5)	9.4 (6)	
Cps2-Plane	C6–C10	20.3 (4)	10.0 (5)	10.1 (6)	1.1 (5)

**Fig. 1.**
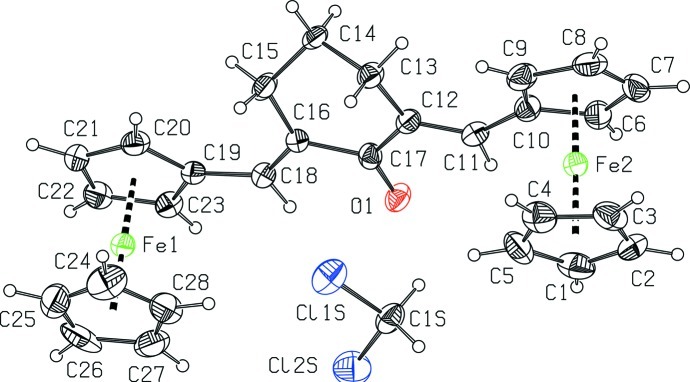
The molecular structure of the title compound, showing 30% probability ellipsoids.

**Fig. 2.**
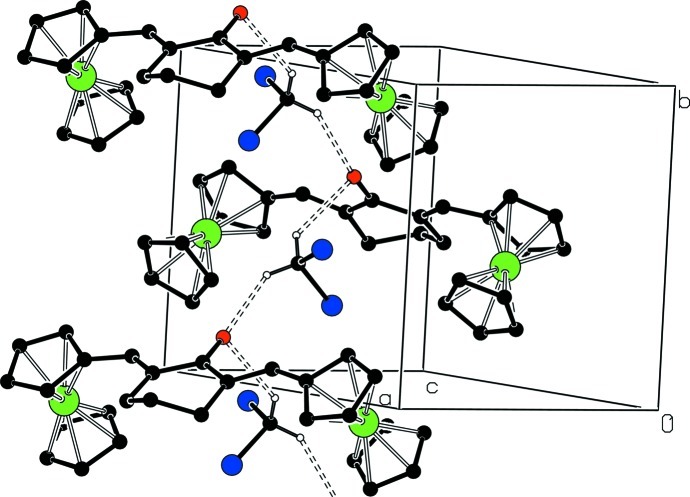
Part of the crystal structure of the title compound, showing the two C—H···O intermolecular hydrogen-bonds linking the molecules into [010] chains. For the sake of clarity, H atoms not involved in hydrogen bonding have been omitted.

## supplementary crystallographic information

### Comment

As part of our ongoing studies of ferrocenyl derivatives (Long *et al.*, 2008; Liu *et al.*, 2008; Liu & Guo, 2010) we now report the structure of the title compound.

The molecule of the title compound (I) exists as the most stable configuration of (*E*,*E*)-isomer (Fig.1). In the two ferrocenyl moieties Fe1 atom is further to the plane of Cps1 (the substituted cyclopentadienyl ring) (Table 1) and nearer to the plane of Cp1 (the unsubstituted cyclopentadienyl ring) but Fe2 atom is nearer to the plane of Cps2 and further to the plane of Cp2, just in reverse manner, with distances of Fe1 to Cg1 and Cgs1 (Cg and Cgs are respectively the centers of Cp and Cps in the every ferroceneyl group) 1.649 (5) Å and 1.641 (4) Å, respectively, and these of Fe2 to Cgs2 and Cg2 1.635 (4) Å and 1.651 (4) Å, respectively. The two planes of Cp and Cps in the every ferrocenyl group are almost parallel because the dihedral angles of Cp1 to Cps1 and Cp2 to Cps2 are 1.1 (5)° and 1.2 (6)° (Table 2), respectively.

The angles of Cgs1—Fe1—Cg1 and Cgs2—Fe2—Cg2 are 177.9 (2) and 179.1 (2)°, respectively. The correlative carbon atoms in Cp1 and Cps1 of ferrocenyl moiety containing Fe1 atom are in an eclipsed conformation but these of containing Fe2 in a slightly cross one because the five pseudo-torsion angles is in the ranges of 4.9 (2)–6.7 (3)° and 22.0 (2)–21.1 (3). All the ferrocenyl data lie in the normal range and just like our previous reported data (Long *et al.*, 2008; Liu *et al.*, 2008; Liu & Guo, 2010).

The conjugated bridge atoms of C11, C12, O1, C16, C17 and C18 are almost co-plane with the plane-1 (determined by perfect co-plane atoms of C12, O1, C16 and C17). The central cyclohexanone ring adopts an safa conformation beause C15 are also co-plane with the plane-1 but C13 and C14 deprivete – 0.101 (8) and 0.669 (8)(8) Å from the plane, respectively.

In the crystal, the molecules are linked by two C—H···O hydrogen-bonds.

### Experimental

Under the protection of argon gas a total of powder potassium hydroxide (1.68 g, 0.03 mol), ferrocenecarboxaldehyde (4.28 g, 0.02 mol) and cyclohexanone (0.98 g, 0.01 mol) were dissolved in 50 ml ethanol and the mixture solution was reacted in a microwave (700 W, generating 2450 MHz frequency) refluxing system for 3 min. Then the red mixture solid was poured into 40 ml water, filtered off, washed with water and and a water/ethanol (1:1) mixture three times. After drying and recrystallization from 95% ethanol, the title compound (4.20 g) was obtained, yield 85.5% and m.p. 436.5–437.8 K (lit. 435.5–436.5; Bai *et al.*, 2004). Orange needles were obtained by slow evaporation of a solution of the solid in dichloromethane/ether (9:1 volume ratio) at room temperature over a period of 8 d.

### Refinement

After their location in a difference map, all H atoms were fixed geometrically at ideal positions and allowed to ride on the parent C atoms, with C—H distances of 0.93 Å (aryl) and 0.97 Å (CH_2_), and with *U*_iso_(H) values of 1.2*U*_eq_(C).

### Crystal data

**Table:**

[Fe_2_(C_5_H_5_)_2_(C_18_H_16_O)]·CH_2_Cl_2_	*F*(000) = 592
*M**_r_* = 575.11	*D*_x_ = 1.528 Mg m^−^^3^
Monoclinic, *P*2_1_	Melting point < 436 K
Hall symbol: P 2yb	Mo *K*α radiation, λ = 0.71073 Å
*a* = 9.417 (4) Å	Cell parameters from 1462 reflections
*b* = 9.330 (4) Å	θ = 2.6–22.3°
*c* = 14.449 (6) Å	µ = 1.40 mm^−^^1^
β = 100.127 (5)°	*T* = 296 K
*V* = 1249.7 (8) Å^3^	Needle, orange
*Z* = 2	0.42 × 0.16 × 0.14 mm

### Data collection

**Table:**

Bruker SMART CCD diffractometer	4224 independent reflections
Radiation source: fine-focus sealed tube	2923 reflections with *I* > 2σ(*I*)
Graphite monochromator	*R*_int_ = 0.036
φ and ω scans	θ_max_ = 25.0°, θ_min_ = 1.4°
Absorption correction: multi-scan (*SADABS*; Sheldrick, 2008*a*)	*h* = −11→10
*T*_min_ = 0.592, *T*_max_ = 0.829	*k* = −11→10
6984 measured reflections	*l* = −17→15

### Refinement

**Table:**

Refinement on *F*^2^	Secondary atom site location: difference Fourier map
Least-squares matrix: full	Hydrogen site location: inferred from neighbouring sites
*R*[*F*^2^ > 2σ(*F*^2^)] = 0.052	H-atom parameters constrained
*wR*(*F*^2^) = 0.127	*w* = 1/[σ^2^(*F*_o_^2^) + (0.0556*P*)^2^ + 0.3275*P*] where *P* = (*F*_o_^2^ + 2*F*_c_^2^)/3
*S* = 1.00	(Δ/σ)_max_ < 0.001
4224 reflections	Δρ_max_ = 0.40 e Å^−^^3^
307 parameters	Δρ_min_ = −0.49 e Å^−^^3^
1 restraint	Absolute structure: Flack (1983), with how many Friedel pairs?
Primary atom site location: structure-invariant direct methods	Flack parameter: 0.60 (4)

### Special details

**Table:**

Geometry. All esds (except the esd in the dihedral angle between two l.s. planes) are estimated using the full covariance matrix. The cell esds are taken into account individually in the estimation of esds in distances, angles and torsion angles; correlations between esds in cell parameters are only used when they are defined by crystal symmetry. An approximate (isotropic) treatment of cell esds is used for estimating esds involving l.s. planes.
Refinement. Refinement of F^2^ against ALL reflections. The weighted R-factor wR and goodness of fit S are based on F^2^, conventional R-factors R are based on F, with F set to zero for negative F^2^. The threshold expression of F^2^ > 2sigma(F^2^) is used only for calculating R-factors(gt) etc. and is not relevant to the choice of reflections for refinement. R-factors based on F^2^ are statistically about twice as large as those based on F, and R-factors based on ALL data will be even larger.

### Fractional atomic coordinates and isotropic or equivalent isotropic displacement parameters (Å^2^)

**Table:**

	*x*	*y*	*z*	*U*_iso_*/*U*_eq_	
C7	0.9697 (9)	0.5905 (10)	0.9230 (5)	0.067 (2)	
H7	1.0320	0.6277	0.9745	0.080*	
C27	0.6739 (10)	0.3833 (11)	0.1317 (8)	0.090 (3)	
H27	0.7451	0.4519	0.1301	0.109*	
Cl1S	0.8503 (2)	0.2625 (3)	0.47016 (17)	0.0799 (7)	
Fe1	0.47321 (9)	0.42146 (10)	0.15974 (6)	0.0430 (3)	
Fe2	1.01841 (9)	0.43903 (11)	0.83256 (5)	0.0446 (3)	
C17	0.6810 (7)	0.5830 (8)	0.5096 (4)	0.0423 (16)	
C20	0.3276 (7)	0.4630 (9)	0.2457 (4)	0.051 (2)	
H20	0.2984	0.4005	0.2889	0.061*	
C21	0.2612 (8)	0.4782 (8)	0.1490 (5)	0.053 (2)	
H21	0.1819	0.4270	0.1184	0.064*	
C18	0.5523 (7)	0.5822 (7)	0.3498 (4)	0.0444 (16)	
H18	0.6333	0.6337	0.3409	0.053*	
C22	0.3371 (8)	0.5839 (8)	0.1088 (5)	0.053 (2)	
H22	0.3166	0.6149	0.0467	0.064*	
C11	0.8029 (6)	0.5823 (7)	0.6702 (4)	0.0436 (16)	
H11	0.8659	0.6411	0.6448	0.052*	
C10	0.8438 (7)	0.5580 (8)	0.7710 (5)	0.0471 (18)	
C19	0.4470 (7)	0.5608 (8)	0.2646 (4)	0.0437 (18)	
C23	0.4501 (8)	0.6353 (9)	0.1785 (5)	0.049 (2)	
H23	0.5159	0.7061	0.1697	0.058*	
C15	0.4325 (6)	0.4580 (9)	0.4690 (4)	0.057 (2)	
H15A	0.3414	0.4910	0.4332	0.068*	
H15B	0.4428	0.3574	0.4547	0.068*	
C4	1.0743 (10)	0.2315 (10)	0.8150 (6)	0.070 (2)	
H4	1.0169	0.1517	0.8196	0.084*	
C3	1.1693 (10)	0.2949 (11)	0.8887 (6)	0.070 (3)	
H3	1.1853	0.2657	0.9512	0.084*	
C14	0.4291 (6)	0.4738 (9)	0.5720 (4)	0.055 (2)	
H14A	0.3556	0.4113	0.5892	0.066*	
H14B	0.4047	0.5718	0.5853	0.066*	
O1	0.7765 (5)	0.6576 (5)	0.4860 (3)	0.0511 (12)	
C28	0.6514 (11)	0.3016 (13)	0.2092 (6)	0.082 (3)	
H28	0.7067	0.3060	0.2692	0.099*	
C2	1.2355 (7)	0.4091 (12)	0.8521 (6)	0.072 (2)	
H2	1.3056	0.4686	0.8855	0.086*	
C5	1.0807 (10)	0.3089 (11)	0.7337 (6)	0.072 (3)	
H5	1.0281	0.2900	0.6742	0.087*	
C25	0.4841 (10)	0.2364 (11)	0.0889 (8)	0.086 (3)	
H25	0.4066	0.1888	0.0532	0.103*	
C1	1.1794 (9)	0.4193 (13)	0.7568 (5)	0.074 (2)	
H1	1.2036	0.4881	0.7157	0.089*	
C6	0.9474 (8)	0.6446 (9)	0.8295 (5)	0.054 (2)	
H6	0.9935	0.7241	0.8096	0.065*	
C13	0.5755 (6)	0.4355 (9)	0.6298 (4)	0.0481 (15)	
H13A	0.5710	0.4422	0.6962	0.058*	
H13B	0.5998	0.3375	0.6165	0.058*	
C16	0.5541 (7)	0.5414 (7)	0.4386 (4)	0.0419 (15)	
C24	0.5346 (12)	0.2143 (11)	0.1820 (8)	0.086 (3)	
H24	0.4962	0.1507	0.2207	0.103*	
C12	0.6901 (6)	0.5347 (7)	0.6072 (4)	0.0417 (15)	
C26	0.5655 (14)	0.3394 (13)	0.0564 (6)	0.091 (4)	
H26	0.5521	0.3747	−0.0047	0.109*	
Cl2S	1.0017 (2)	0.4477 (3)	0.35867 (14)	0.0868 (7)	
C1S	0.9880 (7)	0.3919 (8)	0.4715 (5)	0.059 (2)	
H1S1	0.9676	0.4741	0.5082	0.071*	
H1S2	1.0795	0.3515	0.5016	0.071*	
C9	0.8017 (7)	0.4478 (11)	0.8289 (4)	0.061 (2)	
H9	0.7361	0.3747	0.8096	0.074*	
C8	0.8797 (8)	0.4705 (10)	0.9230 (5)	0.067 (3)	
H8	0.8717	0.4146	0.9752	0.080*	

### Atomic displacement parameters (Å^2^)

**Table:**

	*U*^11^	*U*^22^	*U*^33^	*U*^12^	*U*^13^	*U*^23^
C7	0.057 (5)	0.087 (7)	0.052 (5)	0.026 (5)	−0.002 (4)	−0.020 (5)
C27	0.054 (5)	0.091 (8)	0.134 (9)	−0.013 (5)	0.038 (6)	−0.041 (7)
Cl1S	0.0637 (14)	0.0742 (16)	0.1001 (16)	−0.0055 (12)	0.0095 (12)	0.0086 (13)
Fe1	0.0381 (5)	0.0520 (7)	0.0385 (5)	0.0012 (6)	0.0057 (4)	−0.0013 (6)
Fe2	0.0410 (5)	0.0547 (7)	0.0374 (5)	0.0005 (6)	0.0053 (4)	−0.0011 (6)
C17	0.043 (4)	0.035 (4)	0.046 (4)	0.000 (3)	0.003 (3)	−0.005 (3)
C20	0.044 (4)	0.062 (6)	0.047 (4)	0.005 (4)	0.012 (3)	0.007 (4)
C21	0.042 (4)	0.067 (6)	0.048 (4)	0.000 (4)	0.003 (3)	−0.009 (4)
C18	0.038 (4)	0.050 (4)	0.044 (4)	0.000 (3)	0.004 (3)	−0.002 (3)
C22	0.056 (5)	0.059 (5)	0.043 (4)	0.013 (4)	0.007 (3)	0.006 (4)
C11	0.036 (4)	0.043 (4)	0.052 (4)	0.002 (3)	0.009 (3)	0.007 (3)
C10	0.043 (4)	0.049 (5)	0.048 (4)	0.004 (3)	0.004 (3)	0.002 (3)
C19	0.038 (4)	0.055 (5)	0.039 (4)	−0.002 (3)	0.010 (3)	−0.003 (3)
C23	0.044 (4)	0.051 (5)	0.052 (4)	−0.001 (4)	0.010 (3)	0.003 (4)
C15	0.033 (3)	0.088 (6)	0.048 (3)	−0.014 (4)	0.003 (3)	0.003 (4)
C4	0.079 (6)	0.048 (6)	0.086 (6)	0.003 (5)	0.024 (5)	−0.005 (5)
C3	0.074 (6)	0.086 (7)	0.051 (5)	0.032 (6)	0.011 (4)	0.003 (5)
C14	0.028 (3)	0.096 (6)	0.043 (3)	−0.009 (3)	0.008 (3)	−0.003 (4)
O1	0.041 (3)	0.062 (3)	0.050 (3)	−0.015 (2)	0.005 (2)	0.006 (2)
C28	0.066 (6)	0.125 (9)	0.048 (5)	0.046 (6)	−0.009 (4)	−0.004 (6)
C2	0.036 (4)	0.093 (7)	0.087 (6)	0.000 (5)	0.012 (4)	−0.021 (6)
C5	0.088 (7)	0.076 (7)	0.051 (5)	0.017 (6)	0.005 (4)	−0.021 (5)
C25	0.060 (6)	0.071 (7)	0.120 (9)	0.011 (5)	−0.002 (6)	−0.043 (7)
C1	0.084 (6)	0.075 (6)	0.074 (5)	0.000 (7)	0.044 (4)	0.000 (6)
C6	0.050 (5)	0.052 (5)	0.059 (5)	0.008 (4)	0.002 (4)	−0.008 (4)
C13	0.040 (3)	0.059 (4)	0.045 (3)	−0.003 (4)	0.007 (3)	0.007 (4)
C16	0.037 (4)	0.051 (4)	0.038 (4)	−0.005 (3)	0.008 (3)	−0.007 (3)
C24	0.077 (7)	0.068 (7)	0.113 (9)	0.015 (6)	0.018 (7)	0.017 (6)
C12	0.035 (4)	0.039 (4)	0.051 (4)	0.000 (3)	0.006 (3)	−0.002 (3)
C26	0.124 (9)	0.104 (9)	0.051 (5)	0.056 (7)	0.031 (6)	−0.011 (6)
Cl2S	0.0900 (15)	0.0893 (18)	0.0846 (13)	−0.0011 (16)	0.0246 (11)	0.0082 (15)
C1S	0.040 (4)	0.059 (5)	0.073 (5)	0.006 (4)	−0.002 (3)	0.003 (4)
C9	0.043 (4)	0.088 (6)	0.052 (4)	0.011 (5)	0.004 (3)	0.010 (5)
C8	0.049 (4)	0.100 (8)	0.056 (4)	0.022 (5)	0.019 (3)	0.010 (5)

### Geometric parameters (Å, º)

**Table:**

C7—C8	1.405 (11)	C11—C10	1.458 (8)
C7—C6	1.424 (10)	C11—H11	0.9300
C7—Fe2	2.032 (8)	C10—C6	1.424 (9)
C7—H7	0.9300	C10—C9	1.425 (11)
C27—C28	1.401 (14)	C19—C23	1.429 (9)
C27—C26	1.415 (13)	C23—H23	0.9300
C27—Fe1	2.033 (8)	C15—C14	1.502 (8)
C27—H27	0.9300	C15—C16	1.512 (8)
Cl1S—C1S	1.770 (7)	C15—H15A	0.9700
Fe1—C26	2.007 (8)	C15—H15B	0.9700
Fe1—C25	2.019 (9)	C4—C5	1.390 (11)
Fe1—C24	2.027 (10)	C4—C3	1.395 (11)
Fe1—C23	2.030 (8)	C4—H4	0.9300
Fe1—C22	2.037 (7)	C3—C2	1.386 (13)
Fe1—C28	2.039 (8)	C3—H3	0.9300
Fe1—C20	2.043 (6)	C14—C13	1.524 (8)
Fe1—C19	2.044 (7)	C14—H14A	0.9700
Fe1—C21	2.045 (7)	C14—H14B	0.9700
Fe2—C3	2.020 (8)	C28—C24	1.370 (14)
Fe2—C8	2.025 (7)	C28—H28	0.9300
Fe2—C1	2.028 (7)	C2—C1	1.388 (10)
Fe2—C6	2.029 (8)	C2—H2	0.9300
Fe2—C9	2.034 (6)	C5—C1	1.388 (13)
Fe2—C2	2.034 (7)	C5—H5	0.9300
Fe2—C4	2.034 (9)	C25—C24	1.362 (13)
Fe2—C5	2.038 (8)	C25—C26	1.363 (13)
Fe2—C10	2.053 (7)	C25—H25	0.9300
C17—O1	1.232 (8)	C1—H1	0.9300
C17—C12	1.468 (8)	C6—H6	0.9300
C17—C16	1.484 (8)	C13—C12	1.500 (9)
C20—C21	1.434 (9)	C13—H13A	0.9700
C20—C19	1.436 (9)	C13—H13B	0.9700
C20—H20	0.9300	C24—H24	0.9300
C21—C22	1.402 (10)	C26—H26	0.9300
C21—H21	0.9300	Cl2S—C1S	1.738 (7)
C18—C16	1.335 (8)	C1S—H1S1	0.9700
C18—C19	1.453 (8)	C1S—H1S2	0.9700
C18—H18	0.9300	C9—C8	1.443 (9)
C22—C23	1.414 (9)	C9—H9	0.9300
C22—H22	0.9300	C8—H8	0.9300
C11—C12	1.346 (8)		
			
C8—C7—C6	107.1 (7)	Fe1—C22—H22	126.3
C8—C7—Fe2	69.5 (5)	C12—C11—C10	131.5 (6)
C6—C7—Fe2	69.4 (4)	C12—C11—H11	114.2
C8—C7—H7	126.4	C10—C11—H11	114.2
C6—C7—H7	126.4	C6—C10—C9	107.1 (6)
Fe2—C7—H7	126.3	C6—C10—C11	122.5 (6)
C28—C27—C26	105.5 (9)	C9—C10—C11	130.3 (7)
C28—C27—Fe1	70.1 (5)	C6—C10—Fe2	68.7 (4)
C26—C27—Fe1	68.5 (5)	C9—C10—Fe2	68.9 (4)
C28—C27—H27	127.3	C11—C10—Fe2	124.6 (5)
C26—C27—H27	127.3	C23—C19—C20	106.2 (6)
Fe1—C27—H27	125.7	C23—C19—C18	123.8 (7)
C26—Fe1—C25	39.6 (4)	C20—C19—C18	130.0 (6)
C26—Fe1—C24	66.8 (4)	C23—C19—Fe1	68.9 (4)
C25—Fe1—C24	39.3 (4)	C20—C19—Fe1	69.4 (4)
C26—Fe1—C23	123.0 (4)	C18—C19—Fe1	124.2 (5)
C25—Fe1—C23	157.6 (4)	C22—C23—C19	109.1 (7)
C24—Fe1—C23	161.8 (4)	C22—C23—Fe1	69.9 (4)
C26—Fe1—C27	41.0 (4)	C19—C23—Fe1	70.0 (4)
C25—Fe1—C27	67.5 (4)	C22—C23—H23	125.5
C24—Fe1—C27	67.4 (4)	C19—C23—H23	125.5
C23—Fe1—C27	108.9 (4)	Fe1—C23—H23	126.2
C26—Fe1—C22	110.0 (3)	C14—C15—C16	112.7 (6)
C25—Fe1—C22	122.5 (4)	C14—C15—H15A	109.0
C24—Fe1—C22	155.6 (4)	C16—C15—H15A	109.0
C23—Fe1—C22	40.7 (3)	C14—C15—H15B	109.0
C27—Fe1—C22	127.3 (4)	C16—C15—H15B	109.0
C26—Fe1—C28	67.3 (4)	H15A—C15—H15B	107.8
C25—Fe1—C28	66.2 (4)	C5—C4—C3	107.9 (9)
C24—Fe1—C28	39.4 (4)	C5—C4—Fe2	70.2 (5)
C23—Fe1—C28	126.4 (4)	C3—C4—Fe2	69.3 (5)
C27—Fe1—C28	40.3 (4)	C5—C4—H4	126.0
C22—Fe1—C28	164.1 (4)	C3—C4—H4	126.0
C26—Fe1—C20	161.8 (5)	Fe2—C4—H4	126.0
C25—Fe1—C20	124.8 (4)	C2—C3—C4	107.8 (8)
C24—Fe1—C20	106.7 (4)	C2—C3—Fe2	70.5 (5)
C23—Fe1—C20	68.5 (3)	C4—C3—Fe2	70.4 (5)
C27—Fe1—C20	154.3 (4)	C2—C3—H3	126.1
C22—Fe1—C20	68.4 (3)	C4—C3—H3	126.1
C28—Fe1—C20	119.3 (3)	Fe2—C3—H3	124.6
C26—Fe1—C19	156.7 (5)	C15—C14—C13	110.2 (5)
C25—Fe1—C19	160.6 (4)	C15—C14—H14A	109.6
C24—Fe1—C19	123.9 (4)	C13—C14—H14A	109.6
C23—Fe1—C19	41.1 (3)	C15—C14—H14B	109.6
C27—Fe1—C19	119.8 (3)	C13—C14—H14B	109.6
C22—Fe1—C19	69.2 (3)	H14A—C14—H14B	108.1
C28—Fe1—C19	106.7 (3)	C24—C28—C27	108.7 (8)
C20—Fe1—C19	41.2 (3)	C24—C28—Fe1	69.8 (5)
C26—Fe1—C21	126.0 (4)	C27—C28—Fe1	69.6 (5)
C25—Fe1—C21	108.6 (3)	C24—C28—H28	125.6
C24—Fe1—C21	120.4 (4)	C27—C28—H28	125.6
C23—Fe1—C21	68.3 (3)	Fe1—C28—H28	126.4
C27—Fe1—C21	163.7 (4)	C3—C2—C1	108.2 (9)
C22—Fe1—C21	40.2 (3)	C3—C2—Fe2	69.5 (5)
C28—Fe1—C21	154.3 (4)	C1—C2—Fe2	69.8 (4)
C20—Fe1—C21	41.1 (2)	C3—C2—H2	125.9
C19—Fe1—C21	69.4 (3)	C1—C2—H2	125.9
C3—Fe2—C8	109.0 (3)	Fe2—C2—H2	126.4
C3—Fe2—C1	67.5 (4)	C1—C5—C4	107.9 (8)
C8—Fe2—C1	171.7 (4)	C1—C5—Fe2	69.6 (5)
C3—Fe2—C6	147.6 (4)	C4—C5—Fe2	69.9 (5)
C8—Fe2—C6	68.3 (3)	C1—C5—H5	126.0
C1—Fe2—C6	110.4 (4)	C4—C5—H5	126.0
C3—Fe2—C7	115.3 (3)	Fe2—C5—H5	126.0
C8—Fe2—C7	40.5 (3)	C24—C25—C26	109.1 (10)
C1—Fe2—C7	133.3 (4)	C24—C25—Fe1	70.6 (6)
C6—Fe2—C7	41.0 (3)	C26—C25—Fe1	69.7 (5)
C3—Fe2—C9	132.0 (4)	C24—C25—H25	125.4
C8—Fe2—C9	41.6 (3)	C26—C25—H25	125.4
C1—Fe2—C9	146.2 (3)	Fe1—C25—H25	125.8
C6—Fe2—C9	68.7 (4)	C2—C1—C5	108.1 (9)
C7—Fe2—C9	69.5 (3)	C2—C1—Fe2	70.2 (4)
C3—Fe2—C2	40.0 (4)	C5—C1—Fe2	70.4 (5)
C8—Fe2—C2	132.6 (3)	C2—C1—H1	126.0
C1—Fe2—C2	40.0 (3)	C5—C1—H1	126.0
C6—Fe2—C2	116.9 (4)	Fe2—C1—H1	125.0
C7—Fe2—C2	110.0 (4)	C7—C6—C10	109.5 (7)
C9—Fe2—C2	171.5 (4)	C7—C6—Fe2	69.6 (4)
C3—Fe2—C4	40.3 (3)	C10—C6—Fe2	70.5 (4)
C8—Fe2—C4	115.4 (4)	C7—C6—H6	125.3
C1—Fe2—C4	67.2 (4)	C10—C6—H6	125.3
C6—Fe2—C4	171.3 (3)	Fe2—C6—H6	126.2
C7—Fe2—C4	146.6 (4)	C12—C13—C14	110.6 (6)
C9—Fe2—C4	108.4 (4)	C12—C13—H13A	109.5
C2—Fe2—C4	67.1 (4)	C14—C13—H13A	109.5
C3—Fe2—C5	67.4 (3)	C12—C13—H13B	109.5
C8—Fe2—C5	146.8 (4)	C14—C13—H13B	109.5
C1—Fe2—C5	39.9 (4)	H13A—C13—H13B	108.1
C6—Fe2—C5	133.0 (3)	C18—C16—C17	117.6 (6)
C7—Fe2—C5	172.2 (4)	C18—C16—C15	122.9 (6)
C9—Fe2—C5	114.6 (4)	C17—C16—C15	119.5 (5)
C2—Fe2—C5	67.0 (3)	C25—C24—C28	108.4 (9)
C4—Fe2—C5	39.9 (3)	C25—C24—Fe1	70.0 (6)
C3—Fe2—C10	170.9 (4)	C28—C24—Fe1	70.8 (6)
C8—Fe2—C10	69.1 (3)	C25—C24—H24	125.8
C1—Fe2—C10	115.7 (3)	C28—C24—H24	125.8
C6—Fe2—C10	40.8 (3)	Fe1—C24—H24	125.0
C7—Fe2—C10	69.4 (3)	C11—C12—C17	117.0 (6)
C9—Fe2—C10	40.8 (3)	C11—C12—C13	124.6 (6)
C2—Fe2—C10	147.5 (4)	C17—C12—C13	118.4 (6)
C4—Fe2—C10	131.8 (3)	C25—C26—C27	108.2 (9)
C5—Fe2—C10	109.0 (3)	C25—C26—Fe1	70.7 (5)
O1—C17—C12	121.2 (6)	C27—C26—Fe1	70.5 (5)
O1—C17—C16	119.6 (6)	C25—C26—H26	125.9
C12—C17—C16	119.2 (6)	C27—C26—H26	125.9
C21—C20—C19	108.3 (6)	Fe1—C26—H26	124.5
C21—C20—Fe1	69.5 (4)	Cl2S—C1S—Cl1S	111.7 (4)
C19—C20—Fe1	69.5 (4)	Cl2S—C1S—H1S1	109.3
C21—C20—H20	125.8	Cl1S—C1S—H1S1	109.3
C19—C20—H20	125.8	Cl2S—C1S—H1S2	109.3
Fe1—C20—H20	126.8	Cl1S—C1S—H1S2	109.3
C22—C21—C20	107.9 (7)	H1S1—C1S—H1S2	108.0
C22—C21—Fe1	69.6 (4)	C10—C9—C8	107.4 (8)
C20—C21—Fe1	69.4 (4)	C10—C9—Fe2	70.3 (4)
C22—C21—H21	126.1	C8—C9—Fe2	68.9 (4)
C20—C21—H21	126.1	C10—C9—H9	126.3
Fe1—C21—H21	126.5	C8—C9—H9	126.3
C16—C18—C19	131.9 (6)	Fe2—C9—H9	126.1
C16—C18—H18	114.1	C7—C8—C9	108.9 (7)
C19—C18—H18	114.1	C7—C8—Fe2	70.0 (4)
C21—C22—C23	108.5 (6)	C9—C8—Fe2	69.5 (4)
C21—C22—Fe1	70.2 (4)	C7—C8—H8	125.5
C23—C22—Fe1	69.4 (4)	C9—C8—H8	125.5
C21—C22—H22	125.7	Fe2—C8—H8	126.5
C23—C22—H22	125.7		
			
C28—C27—Fe1—C26	−116.6 (9)	C20—Fe1—C28—C24	80.5 (6)
C28—C27—Fe1—C25	−79.3 (7)	C19—Fe1—C28—C24	123.4 (6)
C26—C27—Fe1—C25	37.3 (6)	C21—Fe1—C28—C24	46.2 (10)
C28—C27—Fe1—C24	−36.5 (6)	C26—Fe1—C28—C27	39.5 (6)
C26—C27—Fe1—C24	80.1 (7)	C25—Fe1—C28—C27	82.7 (6)
C28—C27—Fe1—C23	124.4 (6)	C24—Fe1—C28—C27	120.0 (8)
C26—C27—Fe1—C23	−119.0 (7)	C23—Fe1—C28—C27	−75.7 (6)
C28—C27—Fe1—C22	166.2 (6)	C22—Fe1—C28—C27	−44.0 (13)
C26—C27—Fe1—C22	−77.2 (7)	C20—Fe1—C28—C27	−159.5 (6)
C26—C27—Fe1—C28	116.6 (9)	C19—Fe1—C28—C27	−116.6 (6)
C28—C27—Fe1—C20	45.0 (11)	C21—Fe1—C28—C27	166.3 (7)
C26—C27—Fe1—C20	161.6 (8)	C4—C3—C2—C1	1.6 (10)
C28—C27—Fe1—C19	80.7 (7)	Fe2—C3—C2—C1	−59.2 (7)
C26—C27—Fe1—C19	−162.7 (6)	C4—C3—C2—Fe2	60.9 (6)
C28—C27—Fe1—C21	−158.5 (11)	C8—Fe2—C2—C3	65.8 (8)
C26—C27—Fe1—C21	−41.9 (15)	C1—Fe2—C2—C3	−119.6 (10)
C8—C7—Fe2—C3	−90.0 (5)	C6—Fe2—C2—C3	150.2 (5)
C6—C7—Fe2—C3	151.5 (5)	C7—Fe2—C2—C3	105.9 (6)
C6—C7—Fe2—C8	−118.5 (6)	C9—Fe2—C2—C3	21 (3)
C8—C7—Fe2—C1	−172.1 (5)	C4—Fe2—C2—C3	−38.3 (5)
C6—C7—Fe2—C1	69.4 (6)	C5—Fe2—C2—C3	−81.8 (6)
C8—C7—Fe2—C6	118.5 (6)	C10—Fe2—C2—C3	−171.3 (6)
C8—C7—Fe2—C9	37.8 (4)	C3—Fe2—C2—C1	119.6 (10)
C6—C7—Fe2—C9	−80.7 (5)	C8—Fe2—C2—C1	−174.6 (7)
C8—C7—Fe2—C2	−133.2 (5)	C6—Fe2—C2—C1	−90.2 (7)
C6—C7—Fe2—C2	108.4 (5)	C7—Fe2—C2—C1	−134.5 (7)
C8—C7—Fe2—C4	−54.8 (8)	C9—Fe2—C2—C1	141 (2)
C6—C7—Fe2—C4	−173.2 (6)	C4—Fe2—C2—C1	81.3 (7)
C8—C7—Fe2—C5	161 (2)	C5—Fe2—C2—C1	37.8 (7)
C6—C7—Fe2—C5	43 (3)	C10—Fe2—C2—C1	−51.7 (10)
C8—C7—Fe2—C10	81.5 (5)	C3—C4—C5—C1	0.2 (10)
C6—C7—Fe2—C10	−37.0 (4)	Fe2—C4—C5—C1	59.4 (6)
C26—Fe1—C20—C21	−51.3 (13)	C3—C4—C5—Fe2	−59.3 (6)
C25—Fe1—C20—C21	−78.2 (6)	C3—Fe2—C5—C1	−81.4 (6)
C24—Fe1—C20—C21	−117.4 (6)	C8—Fe2—C5—C1	−171.9 (6)
C23—Fe1—C20—C21	81.2 (5)	C6—Fe2—C5—C1	68.2 (7)
C27—Fe1—C20—C21	170.2 (8)	C7—Fe2—C5—C1	31 (3)
C22—Fe1—C20—C21	37.2 (5)	C9—Fe2—C5—C1	151.3 (5)
C28—Fe1—C20—C21	−158.2 (6)	C2—Fe2—C5—C1	−37.8 (6)
C19—Fe1—C20—C21	119.9 (7)	C4—Fe2—C5—C1	−119.1 (8)
C26—Fe1—C20—C19	−171.2 (10)	C10—Fe2—C5—C1	107.6 (6)
C25—Fe1—C20—C19	161.9 (5)	C3—Fe2—C5—C4	37.7 (6)
C24—Fe1—C20—C19	122.7 (5)	C8—Fe2—C5—C4	−52.8 (9)
C23—Fe1—C20—C19	−38.7 (4)	C1—Fe2—C5—C4	119.1 (8)
C27—Fe1—C20—C19	50.3 (10)	C6—Fe2—C5—C4	−172.7 (6)
C22—Fe1—C20—C19	−82.6 (5)	C7—Fe2—C5—C4	150 (2)
C28—Fe1—C20—C19	81.9 (6)	C9—Fe2—C5—C4	−89.6 (6)
C21—Fe1—C20—C19	−119.9 (7)	C2—Fe2—C5—C4	81.3 (6)
C19—C20—C21—C22	−0.4 (8)	C10—Fe2—C5—C4	−133.3 (6)
Fe1—C20—C21—C22	−59.2 (5)	C26—Fe1—C25—C24	120.0 (9)
C19—C20—C21—Fe1	58.8 (5)	C23—Fe1—C25—C24	166.5 (8)
C26—Fe1—C21—C22	−78.3 (6)	C27—Fe1—C25—C24	81.3 (7)
C25—Fe1—C21—C22	−118.7 (5)	C22—Fe1—C25—C24	−157.7 (6)
C24—Fe1—C21—C22	−160.3 (5)	C28—Fe1—C25—C24	37.4 (6)
C23—Fe1—C21—C22	37.6 (4)	C20—Fe1—C25—C24	−72.8 (7)
C27—Fe1—C21—C22	−45.5 (14)	C19—Fe1—C25—C24	−34.9 (13)
C28—Fe1—C21—C22	167.6 (8)	C21—Fe1—C25—C24	−115.6 (6)
C20—Fe1—C21—C22	119.3 (7)	C24—Fe1—C25—C26	−120.0 (9)
C19—Fe1—C21—C22	81.7 (5)	C23—Fe1—C25—C26	46.5 (11)
C26—Fe1—C21—C20	162.4 (6)	C27—Fe1—C25—C26	−38.7 (6)
C25—Fe1—C21—C20	122.0 (6)	C22—Fe1—C25—C26	82.3 (7)
C24—Fe1—C21—C20	80.4 (6)	C28—Fe1—C25—C26	−82.6 (7)
C23—Fe1—C21—C20	−81.7 (5)	C20—Fe1—C25—C26	167.2 (6)
C27—Fe1—C21—C20	−164.8 (11)	C19—Fe1—C25—C26	−154.9 (9)
C22—Fe1—C21—C20	−119.3 (7)	C21—Fe1—C25—C26	124.4 (6)
C28—Fe1—C21—C20	48.3 (10)	C3—C2—C1—C5	−1.5 (11)
C19—Fe1—C21—C20	−37.6 (5)	Fe2—C2—C1—C5	−60.6 (6)
C20—C21—C22—C23	0.1 (8)	C3—C2—C1—Fe2	59.0 (6)
Fe1—C21—C22—C23	−59.0 (5)	C4—C5—C1—C2	0.8 (11)
C20—C21—C22—Fe1	59.0 (5)	Fe2—C5—C1—C2	60.4 (6)
C26—Fe1—C22—C21	122.5 (6)	C4—C5—C1—Fe2	−59.6 (6)
C25—Fe1—C22—C21	80.3 (6)	C3—Fe2—C1—C2	−37.2 (7)
C24—Fe1—C22—C21	44.8 (10)	C8—Fe2—C1—C2	29 (3)
C23—Fe1—C22—C21	−119.7 (6)	C6—Fe2—C1—C2	107.9 (7)
C27—Fe1—C22—C21	165.4 (5)	C7—Fe2—C1—C2	67.0 (9)
C28—Fe1—C22—C21	−160.2 (10)	C9—Fe2—C1—C2	−170.3 (8)
C20—Fe1—C22—C21	−38.1 (4)	C4—Fe2—C1—C2	−81.0 (7)
C19—Fe1—C22—C21	−82.3 (4)	C5—Fe2—C1—C2	−118.5 (10)
C26—Fe1—C22—C23	−117.8 (6)	C10—Fe2—C1—C2	152.1 (7)
C25—Fe1—C22—C23	−160.0 (5)	C3—Fe2—C1—C5	81.3 (6)
C24—Fe1—C22—C23	164.5 (8)	C8—Fe2—C1—C5	147 (3)
C27—Fe1—C22—C23	−74.8 (6)	C6—Fe2—C1—C5	−133.6 (5)
C28—Fe1—C22—C23	−40.5 (12)	C7—Fe2—C1—C5	−174.5 (5)
C20—Fe1—C22—C23	81.7 (4)	C9—Fe2—C1—C5	−51.7 (10)
C19—Fe1—C22—C23	37.4 (4)	C2—Fe2—C1—C5	118.5 (10)
C21—Fe1—C22—C23	119.7 (6)	C4—Fe2—C1—C5	37.5 (6)
C12—C11—C10—C6	−164.4 (7)	C10—Fe2—C1—C5	−89.4 (6)
C12—C11—C10—C9	19.8 (12)	C8—C7—C6—C10	−0.1 (8)
C12—C11—C10—Fe2	110.4 (7)	Fe2—C7—C6—C10	59.4 (5)
C3—Fe2—C10—C6	159.9 (17)	C8—C7—C6—Fe2	−59.5 (5)
C8—Fe2—C10—C6	80.6 (5)	C9—C10—C6—C7	−0.5 (8)
C1—Fe2—C10—C6	−92.0 (5)	C11—C10—C6—C7	−177.1 (6)
C7—Fe2—C10—C6	37.1 (4)	Fe2—C10—C6—C7	−58.8 (5)
C9—Fe2—C10—C6	119.3 (6)	C9—C10—C6—Fe2	58.4 (5)
C2—Fe2—C10—C6	−58.0 (7)	C11—C10—C6—Fe2	−118.3 (6)
C4—Fe2—C10—C6	−173.5 (5)	C3—Fe2—C6—C7	−53.6 (8)
C5—Fe2—C10—C6	−134.8 (5)	C8—Fe2—C6—C7	37.9 (4)
C3—Fe2—C10—C9	41 (2)	C1—Fe2—C6—C7	−133.4 (5)
C8—Fe2—C10—C9	−38.6 (5)	C9—Fe2—C6—C7	82.9 (5)
C1—Fe2—C10—C9	148.7 (5)	C2—Fe2—C6—C7	−90.1 (5)
C6—Fe2—C10—C9	−119.3 (6)	C4—Fe2—C6—C7	155 (2)
C7—Fe2—C10—C9	−82.1 (5)	C5—Fe2—C6—C7	−172.8 (5)
C2—Fe2—C10—C9	−177.2 (6)	C10—Fe2—C6—C7	120.6 (6)
C4—Fe2—C10—C9	67.2 (6)	C3—Fe2—C6—C10	−174.2 (5)
C5—Fe2—C10—C9	106.0 (5)	C8—Fe2—C6—C10	−82.7 (5)
C3—Fe2—C10—C11	−84.5 (19)	C1—Fe2—C6—C10	106.1 (5)
C8—Fe2—C10—C11	−163.8 (7)	C7—Fe2—C6—C10	−120.6 (6)
C1—Fe2—C10—C11	23.6 (8)	C9—Fe2—C6—C10	−37.7 (4)
C6—Fe2—C10—C11	115.6 (8)	C2—Fe2—C6—C10	149.3 (4)
C7—Fe2—C10—C11	152.7 (7)	C4—Fe2—C6—C10	34 (3)
C9—Fe2—C10—C11	−125.2 (8)	C5—Fe2—C6—C10	66.6 (6)
C2—Fe2—C10—C11	57.6 (9)	C15—C14—C13—C12	61.5 (8)
C4—Fe2—C10—C11	−57.9 (8)	C19—C18—C16—C17	−179.7 (7)
C5—Fe2—C10—C11	−19.2 (7)	C19—C18—C16—C15	−0.7 (11)
C21—C20—C19—C23	0.6 (8)	O1—C17—C16—C18	3.0 (10)
Fe1—C20—C19—C23	59.4 (5)	C12—C17—C16—C18	−177.3 (6)
C21—C20—C19—C18	−176.8 (7)	O1—C17—C16—C15	−176.0 (7)
Fe1—C20—C19—C18	−118.0 (7)	C12—C17—C16—C15	3.7 (9)
C21—C20—C19—Fe1	−58.8 (5)	C14—C15—C16—C18	−157.5 (6)
C16—C18—C19—C23	167.6 (7)	C14—C15—C16—C17	21.4 (9)
C16—C18—C19—C20	−15.4 (13)	C26—C25—C24—C28	−1.3 (12)
C16—C18—C19—Fe1	−106.3 (8)	Fe1—C25—C24—C28	−60.6 (7)
C26—Fe1—C19—C23	55.4 (10)	C26—C25—C24—Fe1	59.3 (7)
C25—Fe1—C19—C23	−167.8 (9)	C27—C28—C24—C25	1.2 (12)
C24—Fe1—C19—C23	166.3 (5)	Fe1—C28—C24—C25	60.2 (7)
C27—Fe1—C19—C23	84.9 (6)	C27—C28—C24—Fe1	−59.0 (7)
C22—Fe1—C19—C23	−37.1 (4)	C26—Fe1—C24—C25	−36.9 (6)
C28—Fe1—C19—C23	126.7 (6)	C23—Fe1—C24—C25	−163.3 (10)
C20—Fe1—C19—C23	−117.7 (6)	C27—Fe1—C24—C25	−81.6 (6)
C21—Fe1—C19—C23	−80.2 (4)	C22—Fe1—C24—C25	50.6 (11)
C26—Fe1—C19—C20	173.0 (8)	C28—Fe1—C24—C25	−118.9 (8)
C25—Fe1—C19—C20	−50.1 (11)	C20—Fe1—C24—C25	125.0 (6)
C24—Fe1—C19—C20	−76.0 (5)	C19—Fe1—C24—C25	166.8 (6)
C23—Fe1—C19—C20	117.7 (6)	C21—Fe1—C24—C25	82.4 (7)
C27—Fe1—C19—C20	−157.4 (5)	C26—Fe1—C24—C28	82.0 (6)
C22—Fe1—C19—C20	80.6 (4)	C25—Fe1—C24—C28	118.9 (8)
C28—Fe1—C19—C20	−115.7 (5)	C23—Fe1—C24—C28	−44.4 (14)
C21—Fe1—C19—C20	37.5 (4)	C27—Fe1—C24—C28	37.3 (5)
C26—Fe1—C19—C18	−61.9 (11)	C22—Fe1—C24—C28	169.5 (6)
C25—Fe1—C19—C18	74.9 (12)	C20—Fe1—C24—C28	−116.1 (6)
C24—Fe1—C19—C18	49.0 (8)	C19—Fe1—C24—C28	−74.3 (6)
C23—Fe1—C19—C18	−117.3 (8)	C21—Fe1—C24—C28	−158.7 (5)
C27—Fe1—C19—C18	−32.4 (8)	C10—C11—C12—C17	179.1 (7)
C22—Fe1—C19—C18	−154.4 (7)	C10—C11—C12—C13	−1.6 (11)
C28—Fe1—C19—C18	9.4 (7)	O1—C17—C12—C11	3.6 (10)
C20—Fe1—C19—C18	125.0 (8)	C16—C17—C12—C11	−176.1 (6)
C21—Fe1—C19—C18	162.5 (7)	O1—C17—C12—C13	−175.7 (7)
C21—C22—C23—C19	0.3 (8)	C16—C17—C12—C13	4.5 (9)
Fe1—C22—C23—C19	−59.2 (5)	C14—C13—C12—C11	144.0 (6)
C21—C22—C23—Fe1	59.5 (5)	C14—C13—C12—C17	−36.7 (8)
C20—C19—C23—C22	−0.5 (8)	C24—C25—C26—C27	0.9 (11)
C18—C19—C23—C22	177.0 (6)	Fe1—C25—C26—C27	60.8 (6)
Fe1—C19—C23—C22	59.1 (5)	C24—C25—C26—Fe1	−59.9 (7)
C20—C19—C23—Fe1	−59.7 (5)	C28—C27—C26—C25	−0.2 (10)
C18—C19—C23—Fe1	117.9 (7)	Fe1—C27—C26—C25	−60.9 (6)
C26—Fe1—C23—C22	82.5 (6)	C28—C27—C26—Fe1	60.7 (6)
C25—Fe1—C23—C22	49.1 (10)	C24—Fe1—C26—C25	36.7 (6)
C24—Fe1—C23—C22	−159.3 (11)	C23—Fe1—C26—C25	−160.7 (6)
C27—Fe1—C23—C22	125.8 (5)	C27—Fe1—C26—C25	118.4 (9)
C28—Fe1—C23—C22	167.2 (5)	C22—Fe1—C26—C25	−117.2 (6)
C20—Fe1—C23—C22	−81.5 (4)	C28—Fe1—C26—C25	79.6 (7)
C19—Fe1—C23—C22	−120.3 (6)	C20—Fe1—C26—C25	−35.7 (14)
C21—Fe1—C23—C22	−37.1 (4)	C19—Fe1—C26—C25	159.1 (8)
C26—Fe1—C23—C19	−157.2 (5)	C21—Fe1—C26—C25	−75.0 (7)
C25—Fe1—C23—C19	169.4 (8)	C25—Fe1—C26—C27	−118.4 (9)
C24—Fe1—C23—C19	−39.0 (13)	C24—Fe1—C26—C27	−81.7 (6)
C27—Fe1—C23—C19	−114.0 (5)	C23—Fe1—C26—C27	80.9 (7)
C22—Fe1—C23—C19	120.3 (6)	C22—Fe1—C26—C27	124.4 (6)
C28—Fe1—C23—C19	−72.5 (6)	C28—Fe1—C26—C27	−38.8 (6)
C20—Fe1—C23—C19	38.8 (4)	C20—Fe1—C26—C27	−154.1 (10)
C21—Fe1—C23—C19	83.2 (4)	C19—Fe1—C26—C27	40.7 (12)
C3—Fe2—C4—C5	−119.1 (8)	C21—Fe1—C26—C27	166.6 (6)
C8—Fe2—C4—C5	151.1 (6)	C6—C10—C9—C8	0.9 (8)
C1—Fe2—C4—C5	−37.5 (5)	C11—C10—C9—C8	177.1 (7)
C6—Fe2—C4—C5	38 (3)	Fe2—C10—C9—C8	59.1 (5)
C7—Fe2—C4—C5	−172.9 (6)	C6—C10—C9—Fe2	−58.2 (5)
C9—Fe2—C4—C5	106.7 (6)	C11—C10—C9—Fe2	118.0 (7)
C2—Fe2—C4—C5	−81.0 (6)	C3—Fe2—C9—C10	−172.0 (5)
C10—Fe2—C4—C5	67.2 (7)	C8—Fe2—C9—C10	118.6 (8)
C8—Fe2—C4—C3	−89.8 (6)	C1—Fe2—C9—C10	−57.3 (10)
C1—Fe2—C4—C3	81.6 (6)	C6—Fe2—C9—C10	37.8 (4)
C6—Fe2—C4—C3	157 (2)	C7—Fe2—C9—C10	81.8 (5)
C7—Fe2—C4—C3	−53.9 (9)	C2—Fe2—C9—C10	170 (2)
C9—Fe2—C4—C3	−134.3 (5)	C4—Fe2—C9—C10	−133.5 (5)
C2—Fe2—C4—C3	38.0 (5)	C5—Fe2—C9—C10	−91.0 (6)
C5—Fe2—C4—C3	119.1 (8)	C3—Fe2—C9—C8	69.3 (7)
C10—Fe2—C4—C3	−173.7 (5)	C1—Fe2—C9—C8	−176.0 (8)
C5—C4—C3—C2	−1.1 (10)	C6—Fe2—C9—C8	−80.9 (6)
Fe2—C4—C3—C2	−60.9 (6)	C7—Fe2—C9—C8	−36.8 (5)
C5—C4—C3—Fe2	59.9 (6)	C2—Fe2—C9—C8	51 (3)
C8—Fe2—C3—C2	−134.8 (5)	C4—Fe2—C9—C8	107.8 (6)
C1—Fe2—C3—C2	37.2 (6)	C5—Fe2—C9—C8	150.4 (6)
C6—Fe2—C3—C2	−55.6 (8)	C10—Fe2—C9—C8	−118.6 (8)
C7—Fe2—C3—C2	−91.4 (6)	C6—C7—C8—C9	0.6 (9)
C9—Fe2—C3—C2	−175.9 (5)	Fe2—C7—C8—C9	−58.8 (5)
C4—Fe2—C3—C2	118.0 (7)	C6—C7—C8—Fe2	59.4 (5)
C5—Fe2—C3—C2	80.6 (6)	C10—C9—C8—C7	−0.9 (9)
C10—Fe2—C3—C2	149.1 (17)	Fe2—C9—C8—C7	59.1 (5)
C8—Fe2—C3—C4	107.2 (6)	C10—C9—C8—Fe2	−60.0 (5)
C1—Fe2—C3—C4	−80.8 (6)	C3—Fe2—C8—C7	107.1 (5)
C6—Fe2—C3—C4	−173.7 (6)	C1—Fe2—C8—C7	44 (3)
C7—Fe2—C3—C4	150.6 (5)	C6—Fe2—C8—C7	−38.4 (4)
C9—Fe2—C3—C4	66.1 (6)	C9—Fe2—C8—C7	−120.3 (7)
C2—Fe2—C3—C4	−118.0 (7)	C2—Fe2—C8—C7	68.8 (7)
C5—Fe2—C3—C4	−37.4 (5)	C4—Fe2—C8—C7	150.2 (5)
C10—Fe2—C3—C4	31 (2)	C5—Fe2—C8—C7	−175.4 (6)
C16—C15—C14—C13	−53.7 (9)	C10—Fe2—C8—C7	−82.4 (5)
C26—C27—C28—C24	−0.6 (11)	C3—Fe2—C8—C9	−132.7 (6)
Fe1—C27—C28—C24	59.1 (7)	C1—Fe2—C8—C9	164 (3)
C26—C27—C28—Fe1	−59.7 (6)	C6—Fe2—C8—C9	81.9 (6)
C26—Fe1—C28—C24	−80.5 (7)	C7—Fe2—C8—C9	120.3 (7)
C25—Fe1—C28—C24	−37.3 (6)	C2—Fe2—C8—C9	−171.0 (7)
C23—Fe1—C28—C24	164.3 (5)	C4—Fe2—C8—C9	−89.5 (6)
C27—Fe1—C28—C24	−120.0 (8)	C5—Fe2—C8—C9	−55.1 (9)
C22—Fe1—C28—C24	−164.0 (10)	C10—Fe2—C8—C9	37.9 (5)

### Hydrogen-bond geometry (Å, º)

**Table:**

*D*—H···*A*	*D*—H	H···*A*	*D*···*A*	*D*—H···*A*
C1*S*—H1*S*1···O1	0.97	2.46	3.210 (9)	133
C1*S*—H1*S*2···O1^i^	0.97	2.25	3.098 (9)	145

Symmetry code: (i) −*x*+2, *y*−1/2, −*z*+1.

### Dihedral angles (°) for selected planes

**Table:**

	Atoms defining plane	1-Plane	Cps1-Plane	Cp1-Plane	Cp2-Plane
1-Plane	O1/C12/C16/C17				
Cps1-Plane	C19–C23	11.2 (4)			
Cp1-Plane	C24–C28	10.5 (6)	1.2 (6)		
Cp2-Plane	C1–C5	19.4 (5)	9.3 (5)	9.4 (6)	
Cps2-Plane	C6–C10	20.3 (4)	10.0 (5)	10.1 (6)	1.1 (5)
